# Natural reversal of cavefish heart asymmetry is controlled by Sonic Hedgehog effects on the left-right organizer

**DOI:** 10.1242/dev.202611

**Published:** 2024-07-18

**Authors:** Mandy Ng, Li Ma, Janet Shi, William R. Jeffery

**Affiliations:** Department of Biology, University of Maryland, College Park, MD 20742, USA

**Keywords:** Cavefish, Heart asymmetry, Sonic Hedgehog, Left-right organizer, *dand5* asymmetry

## Abstract

The direction of left-right visceral asymmetry is conserved in vertebrates. Deviations of the standard asymmetric pattern are rare, and the underlying mechanisms are not understood. Here, we use the teleost *Astyanax mexicanus*, consisting of surface fish with normal left-oriented heart asymmetry and cavefish with high levels of reversed right-oriented heart asymmetry, to explore natural changes in asymmetry determination. We show that Sonic Hedgehog (Shh) signaling is increased at the posterior midline, Kupffer's vesicle (the teleost left-right organizer) is enlarged and contains longer cilia, and the number of dorsal forerunner cells is increased in cavefish. Furthermore, Shh increase in surface fish embryos induces asymmetric changes resembling the cavefish phenotype. Asymmetric expression of the Nodal antagonist Dand5 is equalized or reversed in cavefish, and Shh increase in surface fish mimics changes in cavefish *dand5* asymmetry. Shh decrease reduces the level of right-oriented heart asymmetry in cavefish. Thus, naturally occurring modifications in cavefish heart asymmetry are controlled by the effects of Shh signaling on left-right organizer function.

## Teaser

**Summary:** Natural reversal of cavefish heart asymmetry is controlled by enhanced Sonic Hedgehog signaling at the posterior midline, which modifies left-right organizer function and subsequent *dand5* laterality.

## INTRODUCTION

Bilaterally symmetric animals typically show mirror image symmetry of external organs, such as the eyes and limbs, but also exhibit remarkable asymmetries of internal organs (Blum and Ott, 2018; Blum et al., 2014). In vertebrates, the position of the heart is biased toward the left side of the plane of bilateral symmetry (midline), whereas the liver is mostly located on the right side of the body cavity (Grimes and Burdine, 2017). This pattern of left-right (L-R) visceral asymmetry is conserved among diverse vertebrate species, although low levels of partial or complete inversions have been detected in some natural populations (Malicki et al., 2011; Wang et al., 2011; Noël et al., 2013) or can be induced by mutations in genes governing L-R determination (Chen et al., 2001; Tan et al., 2007).

L-R visceral asymmetry is established during vertebrate embryogenesis through the activity of the L-R organizer (LRO). In amphibians, fish, and some mammals, the LRO is ciliated, and leftward ciliary beating and extracellular fluid flow determine the pattern of visceral organ asymmetry (Blum et al., 2014; Hamada, 2020). The teleost LRO, known as Kupffer's vesicle (KV), is a spherical structure lined by motile cilia (Essner et al., 2005; Kramer-Zucker et al., 2005; Bajoghli et al., 2007; Sampaio et al., 2014; Gokey et al., 2016). The KV develops from the non-involuting dorsal forerunner cells (DFCs), derivatives of the Wilson cells (Warga and Kane, 2018), which converge toward the midline at the leading edge of the blastoderm during embryonic shield formation (Cooper and D'Amico, 1996; Oteíza et al., 2008). Leftward fluid flow reduces expression of the Cerberus/Dan family gene *dand5*, encoding a Nodal antagonist, on the left side, but not the right side, of the KV (Hashimoto et al., 2004; Smith and Uribe, 2021), which in turn promotes expression of the Nodal family gene *spaw* in the left lateral plate mesoderm (LPM) (Long et al., 2003). Spaw subsequently activates the downstream homeobox gene *pitx2*, and the Spaw-Pitx2 signal is propagated from posterior to anterior in the LPM on the left side of the embryo, which determines the L-R pattern of visceral organ asymmetry (Grimes and Burdine, 2017). Spaw is prevented from spreading across the midline by a barrier consisting of BMP4, the Nodal antagonist Lefty1, which is expressed in the notochord, and the Nodal antagonist Lefty2, which is expressed in the left LPM (Bisgrove et al., 1999; Chocron et al., 2007; Lenhardt et al., 2011; Smith et al., 2011). A similar chain of events is initiated in mouse embryos by leftward fluid flow from the LRO resulting in Nodal-Pitx2 activation exclusively in the left LPM, suggesting a conserved sequence of L-R asymmetry determining events in vertebrates with ciliated LROs (Montague et al., 2018; Hamada, 2020; Skenker-Ravi et al., 2022).

Although the mechanisms responsible for establishing L-R visceral asymmetry in vertebrates are fairly well known (Smith and Uribe, 2021), those controlling natural changes in asymmetric patterning are not understood. One of the reasons for this gap in knowledge is that abnormal asymmetry is rare and difficult to study in most vertebrates. For example, heart asymmetry is inverted in only about 1 in 5000-7000 human births (Shiraishi and Ichikawa, 2012). Here, we capitalize on the teleost *Astyanax mexicanus* (Jeffery, 2001, 2020), which provides a unique system for investigating the molecular and evolutionary mechanisms underlying modifications in L-R determination (Ma et al., 2021a).

*Astyanax mexicanus* is a single species with a surface-dwelling form (surface fish; SF) and a cave-dwelling form (cavefish; CF) (Jeffery, 2001, 2020). CF have evolved regressive and constructive traits that differ from SF, including eye degeneration, loss of pigmentation, increased olfactory and taste sensitivity, larger mouthparts and fat deposits, and changes in feeding and social behaviors (Jeffery, 2001, 2020; Xiong et al., 2018; Kowalko, 2020). Many of these traits are accompanied by enhanced embryonic midline structures, namely the pre-chordal plate, notochord, and floor plate of the neural tube, highlighted by upregulation of the Sonic Hedgehog (Shh) signaling system (Yamamoto et al., 2004, 2009; Menuet et al., 2007; Hinaux et al., 2016; Ren et al., 2018; Sifuentes-Romero et al., 2020). Modifications in L-R asymmetry have also evolved in CF: whereas SF, like zebrafish and other vertebrates, show conventional heart asymmetry biased toward the left side of the midline, some CF populations exhibit up to 20-30% right-oriented heart asymmetry (Ma et al., 2021a). Reciprocal hybridization between SF males with normal heart asymmetry and CF females with reversed heart asymmetry has shown that a significant number of the F1 hybrid progeny exhibit the inverted heart asymmetry phenotype of their mothers, indicating that maternal genetic effects are involved in CF L-R asymmetry (Ma et al., 2021a). The laterality of Spaw-Pitx2 signaling in the LPM is also modified in CF (Ma et al., 2021a), suggesting that maternal factors may control L-R asymmetry through unknown zygotic factors acting upstream of Nodal laterality. The purpose of the present investigation was to identify these zygotic factors and understand how they influence L-R asymmetry in CF.

We show that changes in CF heart asymmetry are controlled by upregulation of the Shh pathway at the posterior midline, in a region we term the post-chordal plate. Shh overexpression in the post-chordal plate affects DFC numbers, KV size and ciliation, L-R *dand5* asymmetry, Spaw-Pitx2 laterality in the LPM, and reverses the pattern of L-R heart asymmetry. The results suggest that the evolution of reversed L-R visceral asymmetry in CF is linked to the development of constructive and regressive traits controlled by the Shh midline signaling system.

## RESULTS

### The left-right organizer is modified in CF embryos

**Fig. 1.**
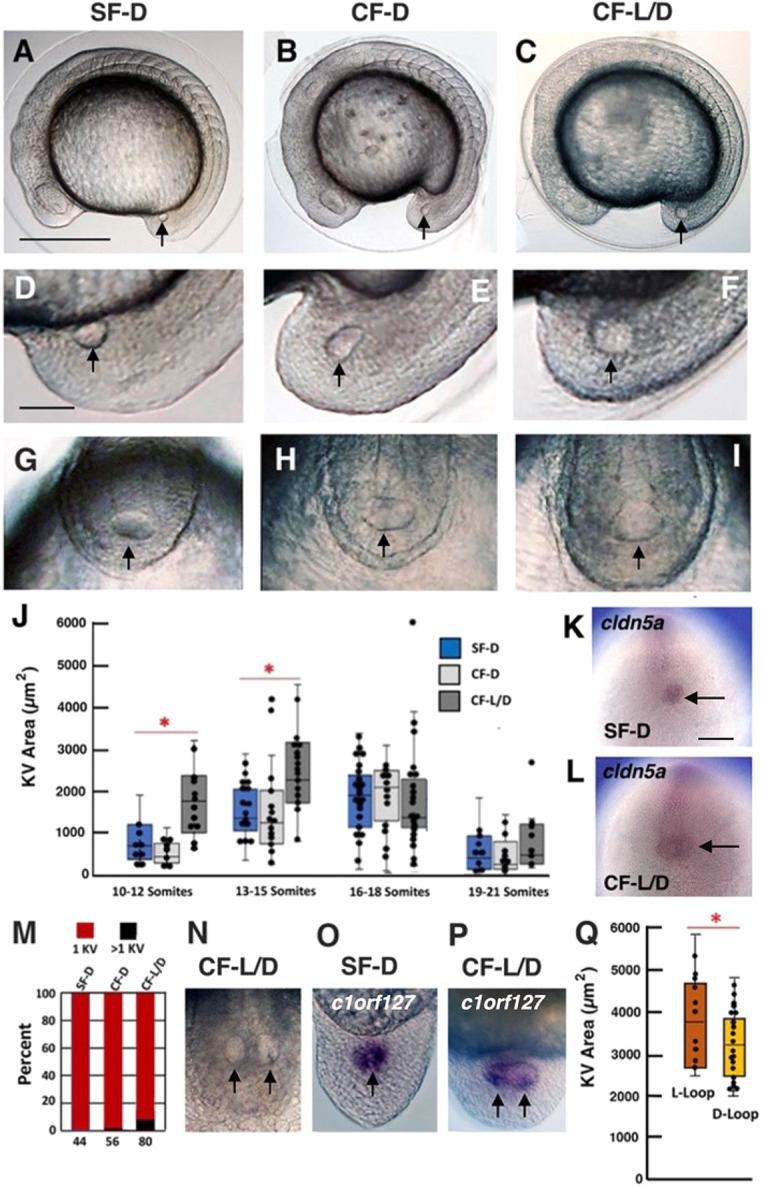
**Left-right organizer modifications and relationship to heart asymmetry in cavefish.** (A-C) SF-D (A), CF-D (B) and CF-L/D (C) embryos at the 13-15 somite stage. (D-I) Tailbud regions of 15-somite SF-D (D,G), CF-D (E,H) and CF-L/D (F,I) embryos viewed from the lateral (D-F) and dorsal-posterior (G-I) sides. (J) KV size differences in SF-D, CF-D and CF-L/D during the 10-12, 13-15, 16-18 and 19-21 somite stages. *P<0.00001 (10-12 somites); *P=0.00141 (13-15 somites). *n*=93 (SF-D), 80 (CF-D) and 79 (CF L-D). (K,L) *In situ* hybridization with the KV lumen marker gene *cldn5a* showing differences in SF-D (K) and CF-L/D (L) KV lumen areas. (M) Bar graph showing the percentage of 15-somite SF-D, CF-D and CF-L/D embryos with multiple KVs. The number of embryos is shown at the base of each bar. (N) A CF-L/D embryo showing two KVs. (O,P) *In situ* hybridization with the KV marker *c1orf127* in 15-somite SF-D (O) and CF-L/D (P) embryos. (Q) Box plot showing the relationship between KV area and the direction of cardiac tube looping in CF-L/D. *P=0.016018. *n*=30. For box and whisker plots in J and Q, box limits represent the interquartile range, horizontal line represents the median, upper and lower whiskers represent the maximum and minimum values, respectively, and dots are individual measurements. Statistical analysis by one-way ANOVA with Tukey HSD. Arrows indicate KVs. Scale bars: 100 µm (in A, for A-C); 30 µm (in D, for D-I,N-P; in K, for K,L).

**Fig. 2.**
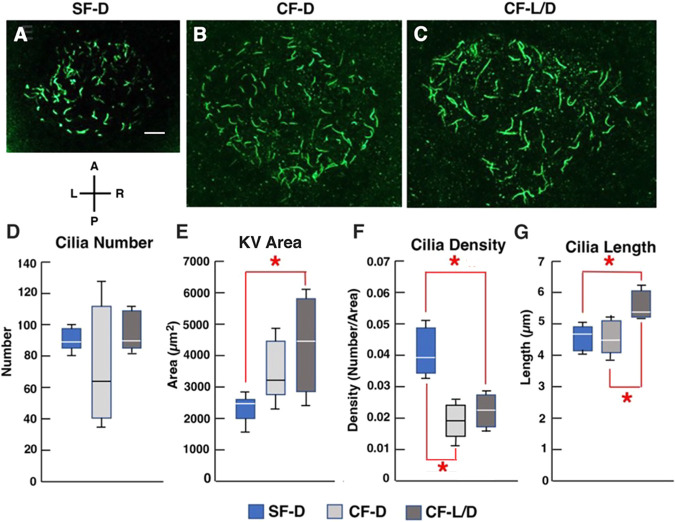
**KV ciliation in surface fish and cavefish.** (A-C) SF-D (A), CF-D (B) and CF-L/D (C) KVs stained with acetylated tubulin antibody at the 15-somite stage. Scale bar: 10 µm (in A, for A-C). (D-G) Box and whisker plots comparing KV cilia number (D), area (E), cilia density (F) and cilia length (G) in 15-somite stage SF-D, CF-D and CF-L/D embryos. Box plot limits represent the interquartile range, horizontal line represents the median, upper and lower whiskers represent the maximum and minimum values, respectively. *n*=9 (SF-D), 5 (CF-D) and 5 (CF L-D). *P=0.00667 (E); *P=0.0075 (F, top); *P=0.00254 (F, bottom); *P=0.00262 (G, top); *P=0.00303 (G, bottom). Statistics by one-way ANOVA with Tukey HSD. A, anterior; L, left; P, posterior; R, right.

To investigate the possibility of differences between SF and CF LROs, we compared KV sizes and ciliation between SF families with >95% D (right)-looping cardiac tubes (SF-D), CF families with 90-95% D-looping cardiac tubes (CF-D), and CF families with 20-30% L (left)-looping cardiac tubes (CF-L/D) (Figs 1 and 2). KV lumen areas were measured from the lateral side at four consecutive stages during embryonic segmentation: the 10-12 somite stage, the 13-15 somite stage, the 16-18 somite stage, and the 19-21 somite stage (Fig. 1A-J). The lumen areas of CF-L/D KVs were significantly larger than SF-D and CF-D KVs between the 10- and 15-somite stages, but KV lumen areas were similar in SF-D and both CF families from the 16- to the 21-somite stages (Fig. 1A-J). Furthermore, *Astyanax* KVs were inferred to inflate and deflate during somitogenesis, as previously shown in zebrafish (Gokey et al., 2016). However, most CF-L/D KV inflated to a larger area than did SF-D or CF-D KVs. The increased area of KV lumens in CF-L/D was confirmed by examining the expression of *claudin5a* (*cldn5a*), a KV lumen marker gene (Kim et al., 2017) (Fig. 1K,L). Surprisingly, a small subset of CF-L/D (about 10%) and even fewer CF-D (about 1-2%) embryos exhibited multiple KVs (Fig. 1M,N): two or three KVs were arranged in a row at the end of a single, posteriorly split notochord (Fig. 1N; also see Fig. 4S), which was not seen in any SF-D embryos (Fig. 1M). The multiple CF KVs were confirmed in CF-L/D embryos by *in situ* hybridization with the KV marker gene *c1orf127* (Fig. 1O,P). The results show that LRO size and number are modified between SF and CF, and that these differences are most pronounced in CF-L/D embryos.

**Fig. 3.**
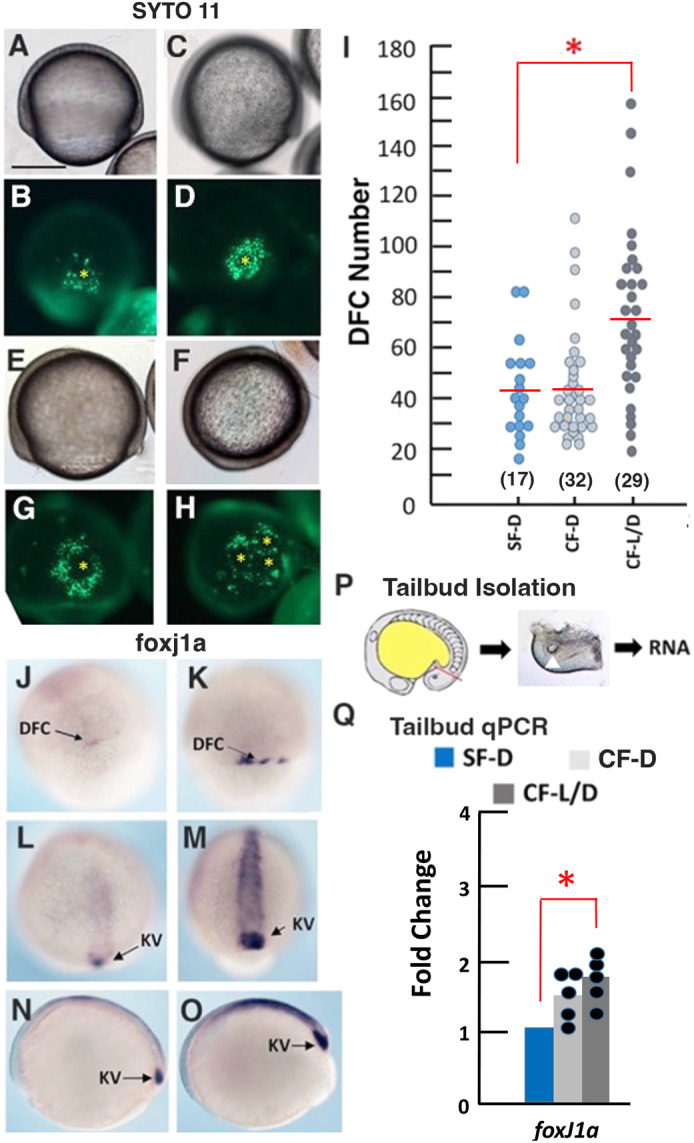
**Increase in dorsal forerunner cells and *foxj1a* expression in cavefish.** (A-H) SYTO 11 staining of dorsal forerunner cells (DFCs) at 70-80% epiboly in SF-D (A,B), CF-D (C,D) and CF-L/D (E-H). A,C,E,F are brightfield images. B,D,G,H are fluorescence images. Yellow asterisks indicate the foci of DFC coalescence. (I) DFC quantification in SF-D, CF-D and CF-L/D embryos at 70-80% epiboly. Red horizontal lines represent the mean. Number of embryos is shown in parentheses. *P=0.000102. Statistics by one-way ANOVA with Tukey HSD. (J-O) *In situ* hybridization showing *foxj1a* expression in SF-D (J,L,N) and CF-L/D (K,M,O) embryos at 50% epiboly (J,K), 70% epiboly (L,M) and early tailbud (N,O) stages. (P) Diagram depicting the isolation of surface fish and cavefish tailbuds for RNA extraction in tailbud qRT-PCR quantifications. Diagonal red line indicates the position of tailbud amputation. Image shows a cavefish tailbud with KV (arrowhead). (Q) Tailbud qRT-PCR quantification of *foxj1a* expression in SF-D, CF-D and CF-L/D embryos at the 13-15 somite stage. *P=0.006802. Statistics by one-way ANOVA with Tukey HSD. *n*=5. Scale bar: 100 µm (in A, for A-H,J-O).

**Fig. 4.**
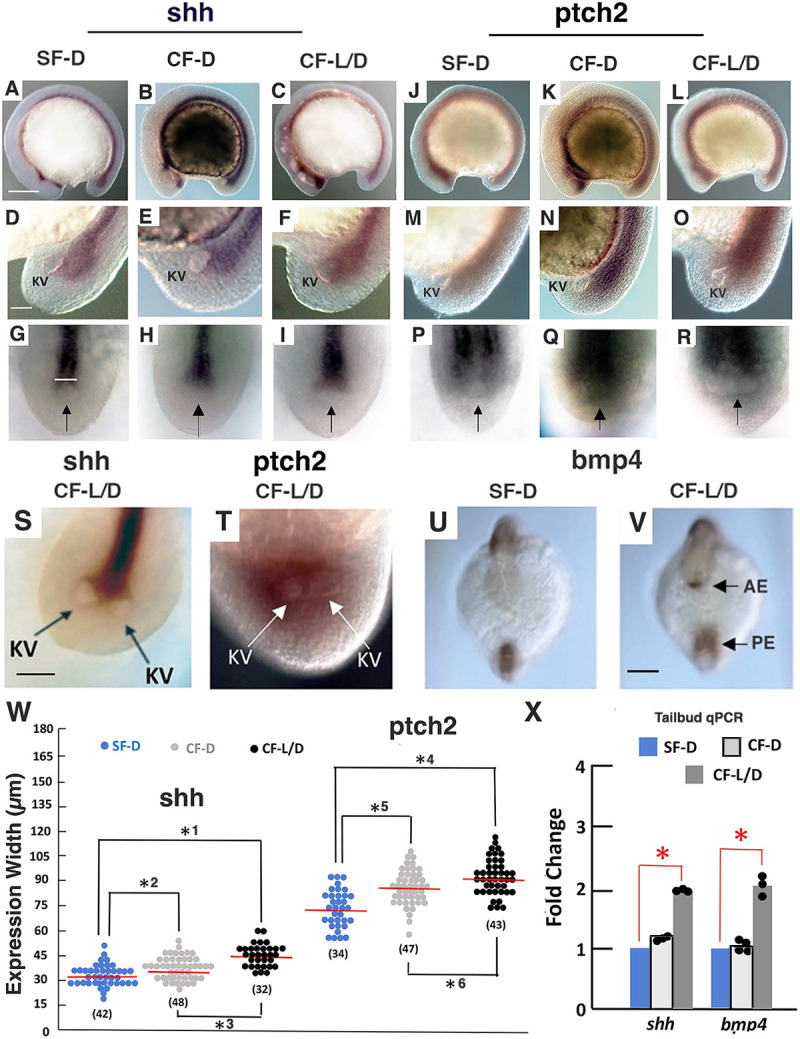
**Shh and BMP signaling is increased along the posterior midline in cavefish.** (A-R) *In situ* hybridization showing *shh* (A-I) and *ptch2* (J-R) expression in 13-15 somite SF-D (A,D,G,J,M,P), CF-D (B,E,H,K,N,Q) and CF-L/D (C,F,I,L,O,R) embryos. Lateral views of embryos (A-C,J-L) and tailbuds (D-F,M-O) and dorsal views of KV (arrows) regions (G-I,P-R). White horizontal line in G indicates width of expression. (S,T) *In situ* hybridization showing *shh* (S) and *ptch2* (T) expression in CF-L/D embryos with two KVs. (U,V) *In situ* hybridization showing *bmp4* expression in 13-15 somite SF-D and CF-L/D embryos. AE, anterior expression domain; PE, posterior expression domain. (W) Widths of *shh* and *ptch2* expression domains in the KV region of SF/D, CF-D and CF-L/D embryos. Red horizontal lines represent means. Number of embryos is shown in parentheses. *^1,3-5^*P*<0.00001; *^2^*P*=0.000857; *^6^*P*=0.000398. Statistics by one-way ANOVA with Tukey HSD. (X) Tailbud qRT-PCR quantification of *shh* and *bmp4* mRNA in 13-15 somite SF-D, CF-D and CF-L/D embryos. *P<0.00001 (*shh*); *P=0.000533 (*bmp4*). *n*=3, 3, 4 and 3 from left to right. Statistics by one-way ANOVA with Tukey HSD. Scale bars: 50 µm (in A, for A-C,J-L; in S, for S,T; in U, for U,V); 30 µm (in D, for D-T).

To determine whether SF and CF KVs differ in ciliation, SF-D, CF-D and CF-L/D embryos were stained with alpha-acetylated tubulin antibody at the 13-15 somite stage, KV lumen areas were measured from the dorsal-posterior pole, and cilia number, density and length were quantified (Fig. 2A-C). The results showed that CF-L/D KVs were significantly larger than SF-D KVs (Fig. 2E). Most CF-D KVs were also larger than SF-D KVs, although the increase did not reach significance (Fig. 2E). No significant differences were found in the number of KV cilia between SF-D, CF-D and CF-L/D embryos (Fig. 2D). Thus, owing to larger KV lumen areas, the density of cilia was significantly reduced, revealing wider spacing between cilia in the KVs of CF-D and CF-L/D embryos compared with those of SF-D embryos (Fig. 2A-C,E,F). Lastly, ciliary length was significantly increased in CF-L/D KVs relative to CF-D and SF-D KVs (Fig. 2G). These results indicate that KV size and ciliation are modified in CF relative to SF embryos, and that the KV modifications are most extreme in CF-L/D embryos.

The relationship between KV modification and the direction of heart asymmetry was determined by measuring the KV sizes of individual 13-15 somite CF-L/D embryos, raising each embryo separately, and then assaying for cardiac tube looping at about 2.5 days post-fertilization (dpf). The results showed that CF-L/D larvae with L-looping heart tubes originated mostly from embryos with significantly larger KVs than CF-L/D larvae with D-looping cardiac tubes (Fig. 1Q). Thus, heart laterality is correlated with KV modifications.

### Dorsal forerunner cells and *foxj1a* expression are increased in CF embryos

We next sought to understand the causes of morphological differences between SF and CF KVs. The KV is formed by coalescence of non-involuting DFCs along the dorsal midline at the shield stage (Cooper and D'Amico, 1996; Oteíza et al., 2008). The DFCs of SF-D, CF-D and CF L/D embryos were stained with SYTO 11, a vital dye that specifically labels highly endocytotic DFCs (Cooper and D'Amico, 1996), and quantified at 70-80% epiboly (Fig. 3A-I). As in zebrafish (Moreno-Ayala et al., 2021), DFC numbers varied greatly within *Astyanax* SF-D, CF-D and CF-L/D embryos (Fig. 3I). However, the mean DFC number was significantly larger in CF-L/D relative to CF-D and SF-D embryos (mean DFC number was not significantly different in CF-D and SF-D embryos) (Fig. 3I). The DFCs of SF-D (Fig. 3A,B), CF-D (Fig. 3C,D), and most CF-L/D embryos (Fig. 3E,G) coalesced into a single focal point during gastrulation, whereas in a subset of CF-L/D embryos DFC formed several focal points (Fig. 3F,H), a likely explanation for the development of multiple KVs (Fig. 1M,N,P). These results suggest that the enlarged and multiple KVs of CF-L/D embryos could be explained by more DFC precursors.

DFCs, and subsequently the KV, express *foxj1a*, the master regulator of motile cilia development (Yu et al., 2008; Tavares et al., 2017). Therefore, we conducted *in situ* hybridization and quantitative reverse transcriptase polymerase chain reaction (qRT-PCR) to determine whether *foxj1a* expression is different in SF and CF embryos (Fig. 3J-Q). *In situ* hybridization showed that *foxj1a* staining is stronger in the DFCs of CF-L/D than SF-D during gastrulation (Fig. 3J,K) and along the midline and in the KV during the 13-15 somite stage (Fig. 3L-O). To quantify *foxj1a* expression specifically in the KV region, we performed qRT-PCR with RNA extracted from isolated tailbuds (Fig. 3P). Tailbud-specific qRT-PCR showed a significant increase in *foxj1a* mRNA levels in CF-L/D embryos compared with SF-D embryos, and *foxj1a* mRNA levels showed a smaller (but not significant) increase in CF-D compared with SF-D embryos (Fig. 3Q). These results suggest that increases in *foxj1a* expression and DFCs may account for structural modifications in the LRO of CF-L/D embryos.

### Sonic Hedgehog and Bone Morphogenetic Protein signaling is increased in the KV region of CF embryos

The *foxj1a* gene is a direct target of Shh signaling (Cruz et al., 2010). Furthermore, previous studies showed that Shh signaling is expanded in the pre-chordal plate at the anterior midline of CF compared with SF embryos and that this increase contributes to lens apoptosis, eye degeneration, and other phenotypic changes in CF (Yamamoto et al., 2004, 2009; Menuet et al., 2007). Accordingly, we hypothesized that increased Shh signaling may also be responsible for KV modifications at the posterior midline. To investigate this possibility, the expression of *shh* and its autoregulated receptor *ptch2* were compared in the KV regions of 13-15 somite SF-D, CF-D and CF-L/D embryos by *in situ* hybridization and qRT-PCR (Fig. 4). We found that *shh* and *ptch2* expression is expanded in the KV regions of CF relative to SF embryos and that this increase is largest in CF-L/D embryos (Fig. 4A-R,W). Furthermore, when two KVs were present in CF-L/D embryos, *shh* expression was associated with spurs of the notochord extending to each of them (Fig. 4S), and expanded *ptch2* expression surrounded both KVs (Fig. 4T). Tailbud-specific qRT-PCR confirmed that *shh* expression was increased in the KV region of CF-L/D, but not CF-D, relative to SF-D embryos (Fig. 4X).

Along with Shh signaling, BMP4 signaling is also expanded in the pre-chordal plate of CF embryos (Pottin et al., 2011; Hinaux et al., 2016). Therefore, we next investigated *bmp4* expression to see whether molecular changes in the KV region resembled those in the pre-chordal plate. *In situ* hybridization showed that *bmp4* expression is also expanded along the posterior midline surrounding the KV in CF-L/D compared with SF-D embryos (Fig. 4U,V), and the increase in *bmp4* expression in the CF-L/D KV region was confirmed by tailbud-specific qRT-PCR (Fig. 4X). These results show that Shh and BMP4 signaling are enhanced in a posterior signaling center surrounding the CF-L/D KV, which we have named the post-chordal plate.

### Sonic Hedgehog increase affects the left-right organizer, eye development, and heart asymmetry in SF

**Fig. 5.**
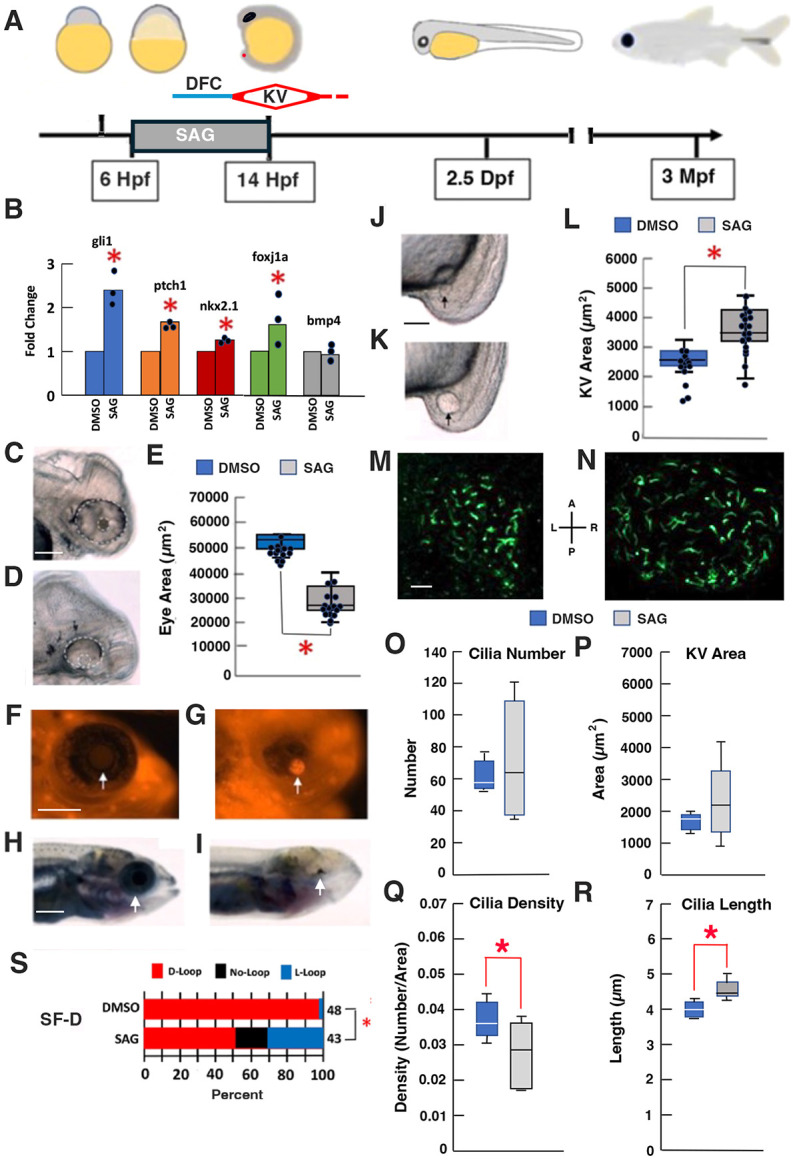
**Effects of increased Sonic Hedgehog expression on KV size and ciliation, eye development, and cardiac looping asymmetry.** (A) The timeline of SAG treatment in surface fish. SAG or DMSO (SAG control) were added to SF-D embryos at 50% epiboly. After washing into water without SAG or DMSO, at the 13-15 somite stage samples were removed for KV size determination and RNA isolation, at 2.5 days post-fertilization (Dpf) for eye measurements, lens apoptosis detection, and cardiac looping assays, and at 3 months post-fertilization (Mpf) for determination of effects on long term optic development. Hpf, hours post-fertilization. Yellow in schematics represents the yolk. (B) Effects of SAG on the expression of Shh signaling genes, *foxj1a* and *bmp4* determined by qRT-PCR at the 13-15 somite stage. *P<0.0001 (*gli1*); *P<0.0001 (*ptch1*); *P=0.001094 (*nkx2.1*); *P=0.038832 (*foxj1a*). Statistics by one-way ANOVA with Tukey HSD. *n*=3 for each fold change. (C-I) Effects of SAG on optic development at 2.5 Dpf (C-E) and 3 Mpf (F-I). (C,D) Reduction of the ventral optic cup in SAG-treated (D) but not control (C) embryos. Eyes and lenses are outlined by white dashed lines. (E) Box and whisker plots showing eye size reduction by SAG. Box limits represent the interquartile range, horizontal line represents the median, upper and lower whiskers represent the maximum and minimum values, respectively, and dots are individual measurements. *P=0.00052. *n*=14. Statistics by one-way ANOVA with Tukey HSD. (F,G) Lens apoptosis in SAG-treated (G) but not control embryos (F). Arrows indicate lenses. (H,I) Eye degeneration in larvae that developed from SAG-treated (I) but not control (H) embryos. Arrows indicate eyes. (J-L) Effects of SAG on KV size. KV size is increased in SAG-treated (K) compared with control (J) embryos at the 13-15 somite stage. Arrows indicate KVs. (L) Box and whisker plots showing increased KV size in SAG compared with control embryos. Graph components are as described for E. *P=0.000031. *n*=15. Statistics by one-way ANOVA with Tukey HSD. (M-R) Effects of SAG on KV ciliation. (M,N) KVs from control (M) and SAG-treated (N) embryos stained with alpha-acetylated tubulin antibody at the 15-somite stage. A, anterior; L, left; P, posterior; R, right. (O-R) Box plots showing KV cilia number (O), area (P), cilia density (Q) and cilia length (R) in 15-somite stage control and SAG-treated embryos. Box limits represent the interquartile range, horizontal line represents the median, upper and lower whiskers represent the maximum and minimum values, respectively. *n*=5 (control) and 7 (SAG). *P=0.036914 (Q); *P=0.001206 (R). Statistics by one-way ANOVA with Tukey HSD. (S) SAG increases cardiac D-looping in SF-D embryos. *P=0.000015. χ^2^ statistic=22.2448. Statistics by χ^2^ test. Scale bars: 100 µm (in C, for C,D; in H, for H,I); 20 µm (in F, for F,G); 30 µm (in J, for J,K); 10 µm (in M, for M,N).

To determine the effects of increased Shh signaling on KV development and heart asymmetry, we treated SF-D embryos with the Smoothened agonist SAG, which is effective in increasing Shh signaling in vertebrate systems (Chen et al., 2002a,b; Lewis and Krieg, 2014; Negretti et al., 2022). In a typical experiment, SAG or DMSO (control) treatment began at 50-60% epiboly and lasted for 8 h (Fig. 5A). Control and SAG-treated embryos were removed for KV measurements, KV cilia staining, and mRNA quantification at the 13-15 somite stage, and at later stages to determine the effects of increased Shh on optic development (Yamamoto et al., 2004, 2009). As shown by qRT-PCR, SAG treatment significantly increased *ptch1*, *gli1*, *nkx2.1* and *foxj1a* mRNA levels relative to DMSO controls and in contrast to the control gene *bmp4* (Fig. 5B), suggesting enhanced Shh signaling and *foxj1a* upregulation. Later in development, the optic primordium was ventrally reduced (Fig. 5C-E), the lens showed apoptosis (Fig. 5F,G), and SAG-treated but not control SF-D larvae developed extremely regressed eyes (Fig. 5H,I). Together, these results suggest that SAG is effective in increasing Shh signaling and producing degenerative eye phenotypes resembling CF. The SAG-treated SF-D embryos showed larger KVs compared with controls at the 13-15 somite stage (Fig. 5J-L), including a small subset with multiple KVs. Furthermore, SAG treated SF-D embryos showed KV ciliation similar to that of CF, including an increase in the length and a decrease in the density of KV cilia compared with controls (Fig. 2; Fig. 5M-R). Lastly, increased levels of cardiac L-looping were seen in SAG-treated SF-D embryos compared with controls (Fig. 5S). These results suggest that pharmacological increase in Shh impacts the size and ciliation of the KV. To complement the long exposure time, SAG treatment was also carried out for shorter (4 h) periods, from 50-60% to 90% epiboly or from 90% epiboly to the 13-15 somite stage (Fig. S1). The shorter treatments showed that heart looping asymmetry was most sensitive to SAG from about 90% epiboly to the 13-15 somite stage (Fig. S1), consistent with the timeline of DFC development and KV expansion (Fig. 5A). Therefore, increased Shh signaling is correlated with modifications in KV development and reversals in the normal pattern of L-R heart asymmetry in SF.

### Left-right *dand5* asymmetry is modified in CF

**Fig. 6.**
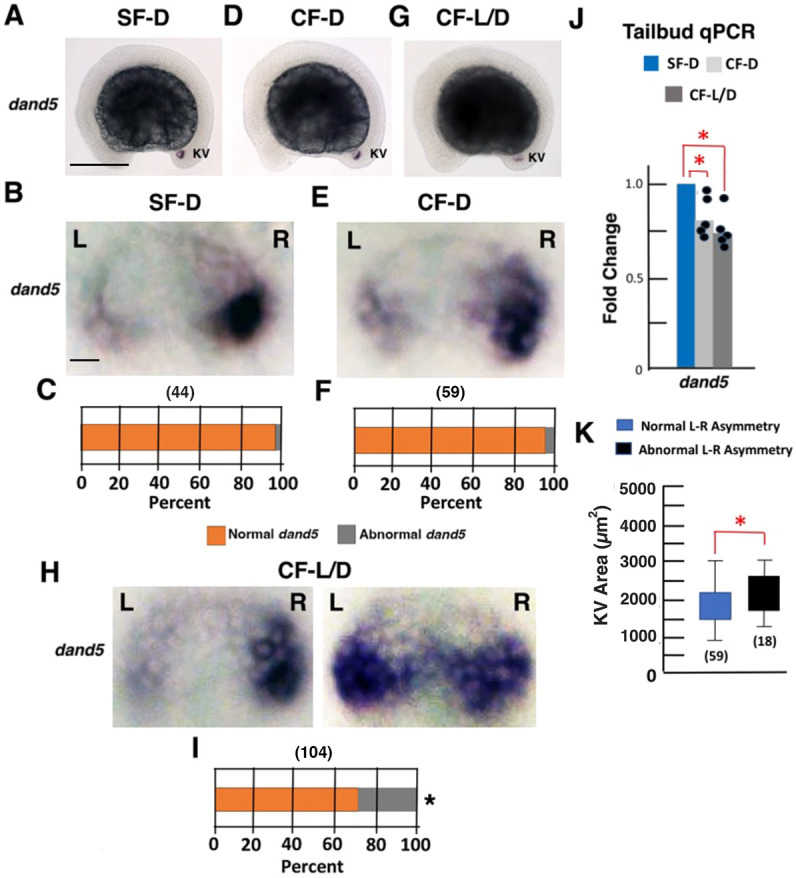
**Left-right asymmetry of *dand5* expression is modified in cavefish.** (A,B,D,E,G,H) *In situ* hybridization showing *dand5* expression in 13-15 somite SF-D (A,B), CF-D (D,E) and CF-L/D (G,H) embryos. (C,F,I) Bar graphs showing the percentage of SF-D (C), CF-D (F) and CF-L/D (I) embryos with normal or abnormal *dand5* L-R asymmetry. Number of embryos shown in parentheses on the top of each bar. L, left; R, right. *P<0.00001. χ^2^ statistic=25.9992. Statistics by χ^2^ test comparing data in C, F and I. (J) Tailbud qRT-PCR quantification of *dand5* mRNA levels in 13-15 somite embryos. *P=0.00150 (top); *P=0.01538 (bottom). Statistics by one-way ANOVA with Tukey HSD. *n*=5. (K) Box plots showing the relationship between *dand5* asymmetry and KV size in 13-15 somite CF-L/D embryos. Box limits represent the interquartile range, and upper and lower whiskers represent the maximum and minimum values, respectively. *P=0.007395. Statistics by one-way ANOVA with Tukey HSD. Number of embryos is shown in parentheses at the base of each bar. Scale bars: 100 µm (in A, for A,D,G); 10 µm (in B, for B,E,H).

L-R determination involves asymmetric expression of the Nodal antagonist Dand5, which is caused by degradation of *dand5* mRNA on the left side of the LRO and activation of *spaw* expression in the left LPM (Marques et al., 2004; Lopes, et al., 2010; Montague et al., 2018; Maerker et al., 2021; Minegishi et al., 2021). Therefore, we next compared *dand5* expression in 13-15 somite SF-D, CF-D and CF-L/D embryos (Fig. 6). *In situ* hybridization showed that *dand5* was expressed exclusively in the KV region at this stage of development (Fig. 6A,D,G). In SF-D embryos, stronger *dand5* staining was detected on the right side of the KV beginning at the 12-13 somite stage (Fig. 6B,C; Fig. S2), indicative of normal L-R *dand5* asymmetry (Montague et al., 2018). Most CF-D and some CF-L/D embryos also exhibited preferential *dand5* staining on the right side of the KV (Fig. 6E,F,H,I). In contrast, *dand5* was expressed abnormally (approximately symmetric or reversed L-R asymmetry) in the KVs of a significant number of CF-L/D embryos (Fig. 6H,I). Tailbud-specific qRT-PCR indicated that *dand5* mRNA levels were significantly decreased in 13-15 somite CF-D and CF-L-D embryos compared with SF-D embryos, with the highest levels of *dand5* mRNA reduction in CF-L/D embryos (Fig. 6J). These results show that normal L-R *dand5* asymmetry is equalized or reversed and expression is downregulated in the KVs of CF-L/D embryos.

To determine whether LRO size and changes in *dand5* asymmetry are correlated, we compared KV sizes in 13-15 somite CF-L/D embryos with normal and abnormal L-R *dand5* asymmetry. The results showed that embryos with larger KVs tended to have abnormal L-R *dand5* asymmetry, whereas embryos with smaller KVs showed normal L-R *dand5* asymmetry (Fig. 6K). These results suggest that development of a larger KV increases the likelihood of changing the patterns of L-R *dand5* expression and asymmetry in CF-L/D embryos.

### Sonic Hedgehog increase affects left-right *dand5* asymmetry in SF

**Fig. 7.**
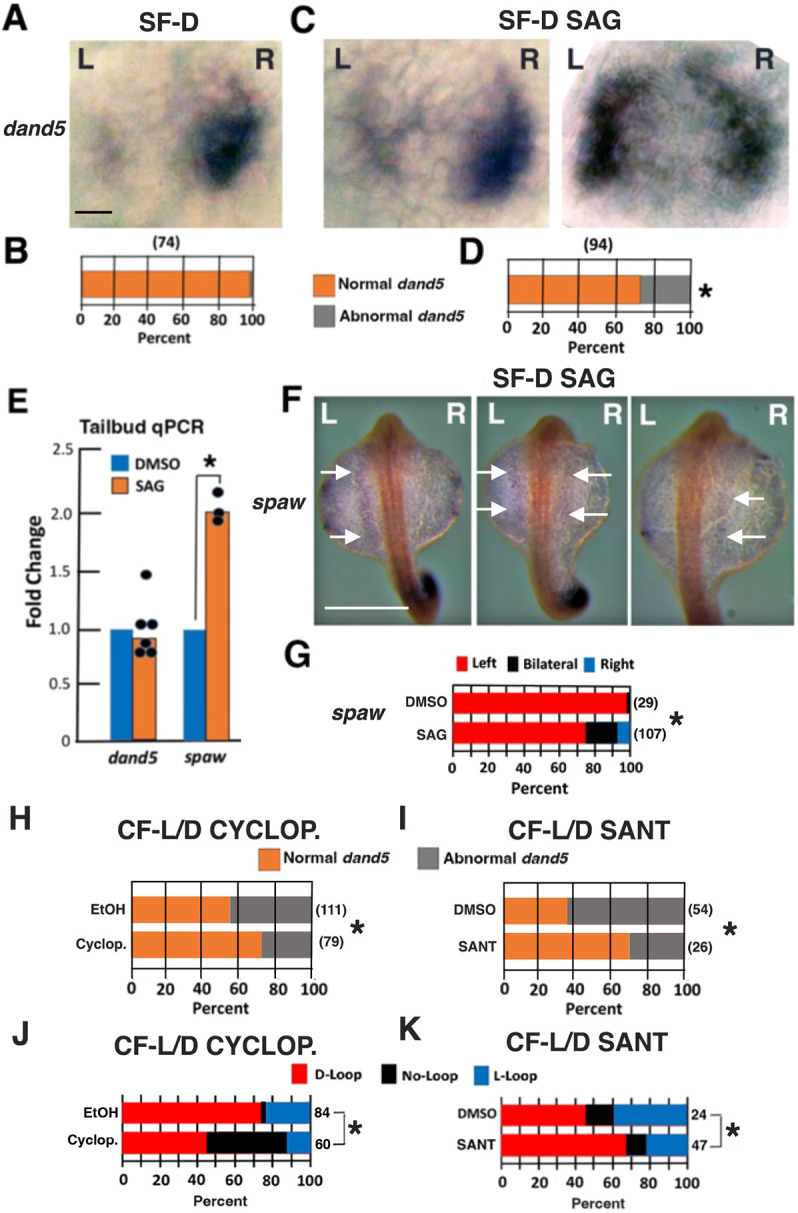
**Sonic Hedgehog increase in surface fish or decrease in cavefish affects *dand5* asymmetry and heart laterality.** (A,C) *In situ* hybridization showing *dand5* expression in 13-15 somite control (A) and SAG-treated (C) SF-D embryos. L, left; R, right. (B,D) Bar graphs showing the percentage of control (B) and SAG-treated (D) SF-D embryos with normal and abnormal *dand5* L-R asymmetry. Number of embryos is shown in parentheses on the top of each bar. *P=0.057275. χ^2^ statistic=3.61156. Statistics by χ^2^ test for data in B and D. (E) Tailbud qRT-PCR quantification of *dand5* (left) and *spaw* (right) mRNA levels in 13-15 somite control and SAG-treated SF-D embryos. *P<0.00001. Statistics by one-way ANOVA with Tukey HSD. *n*=6 and 3 from left to right. (F) *In situ* hybridization showing *spaw* expression (arrows) in the left lateral plate mesoderm (LPM) (left), both the left and right LPM (middle), and the right LPM (right) in 25-somite SAG-treated SF-D embryos. (G) Bar graph quantifying the data in F. Number of embryos is shown in parentheses on the right of each bar. *P=0.035437. χ^2^ statistic=6.6243. Statistics by χ^2^ test. (H-K) Bar graphs showing the effects of cyclopamine (Cyclop.; H,J) and SANT-1 (I,K) on *dand5* (H,I) and cardiac looping (J,K) asymmetry in CF-L/D. EtOH, ethanol control. *P=0.02552 and χ^2^ statistic=4.9883 (H); *P=0.004229 and χ^2^ statistic=8.1829 (I); *P=0.000013 and χ^2^ statistic=22.5168 (J); *P<0.10 and Fisher Exact test statistic=0.0845 (K). Statistics by χ^2^ test in H-J and Fisher Exact test in K. Scale bars: 10 µm (in A, for A,C); 100 µm (F).

To determine whether Shh signaling is involved in changing *dand5* L-R asymmetry, SF-D embryos were treated with SAG for 8 h as described above and the effects on *dand5* expression in the KV region were determined relative to controls (Fig. 7A-E). *In situ* hybridization showed that SAG-treated SF-D embryos, but not controls, developed significantly higher levels of abnormal *dand5* staining (Fig. 7A-D), which resembled modified *dand5* expression in CF-L/D embryos (Fig. 6H,I). Furthermore, tailbud-specific qRT-PCR indicated that *dand5* expression levels were lower in the KV region of SAG-treated SF-D embryos relative to controls (Fig. 7E, left), also similar to CF-L/D embryos (Fig. 6J). These results suggest that abnormal L-R *dand5* asymmetry is mediated by increased Shh signaling.

To address whether Shh increase affects Nodal signaling laterality in the LPM, *spaw* expression was examined in SAG-treated and control embryos (Fig. 7E right, G). *In situ* hybridization showed that SF-D expressed *spaw* only in the left LPM, as described previously (Ma et al., 2021a), whereas in SAG-treated 25-somite SF-D embryos *spaw* expression was detected in the left LPM, in the right LPM, or bilaterally in both LPMs (Fig. 7F,G). The number of SAG-treated SF-D embryos with reversed or bilateral *spaw* expression was similar to that reported previously in CF-L/D embryos (Ma et al., 2021a). In addition, tailbud qPCR showed that *spaw* expression was significantly increased in SAG-treated compared with control SF-D. We conclude that higher Shh expression affects the asymmetry of *dand5* expression in the KV.

### Sonic Hedgehog inhibition affects left-right *dand5* and cardiac looping asymmetry in CF

We next addressed the effects of Shh downregulation on L-R asymmetry in CF. CF-L/D embryos were treated with cyclopamine or SANT-1, which inhibit Shh signaling by binding to Smoothened (Chen et al., 2002a, b), during the same 8 h period as SAG treatment (Fig. 5A). We found that cyclopamine or SANT-1 significantly reduced the levels of abnormal *dand5* mRNA L-R asymmetry, increasing the proportion of CF-L/D embryos with normal *dand5* asymmetry compared with controls (Fig. 7H,I). In addition, both Shh inhibitors significantly decreased the proportion of L-looping cardiac tubes in CF-L/D (Fig. 7J,K). We conclude that normal levels of L-R *dand5* and heart asymmetry can be partially rescued by Shh downregulation in CF-L/D.

## DISCUSSION

We used the teleost *A. mexicanus* to explore the mechanisms underlying natural changes in the highly conserved pattern of L-R visceral organ asymmetry, which is difficult to study in other vertebrates. Features related to midline development and L-R asymmetry determination were compared in SF exhibiting more than 95% normal left-oriented heart asymmetry (SF-D), CF showing highly left-biased heart asymmetry resembling SF (CF-D), and CF showing 20-30% natural reversals of normal heart asymmetry (CF-L/D) (Ma et al., 2021a). The results revealed that changes in LRO form and function related to the natural reversal of L-R heart laterality in CF-L/D embryos is controlled by an enhanced Shh signaling center at the posterior end of the notochord, a region we term the post-chordal plate.

**Fig. 8.**
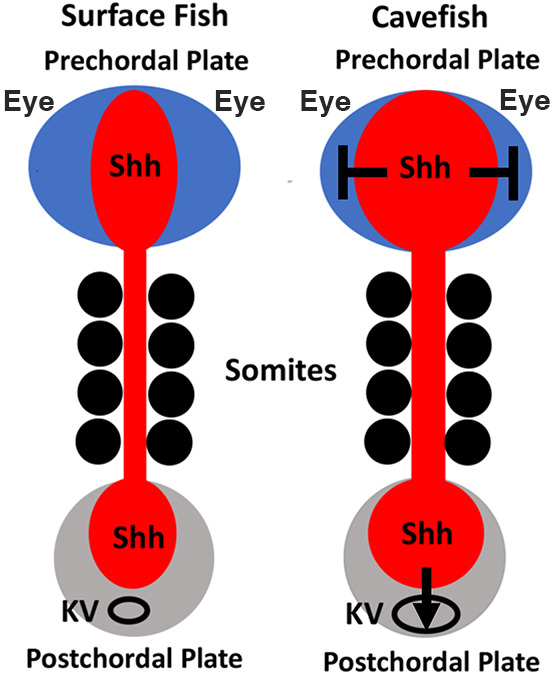
**A model for the effects of Sonic Hedgehog signaling in the surface fish and cavefish pre-chordal and post-chordal plates.** Sonic Hedgehog signaling is expanded in the cavefish pre-chordal plate inducing eye regression and in the post-chordal plate inducing changes in the KV.

Previous studies have shown that enhanced Shh signaling in the pre-chordal plate contributes to eye degeneration, increased olfactory and oral traits, and modified brain development in CF embryos (Yamamoto et al., 2004, 2009; Menuet et al., 2007; Pottin et al., 2011). Here, we show that Shh signaling is also expanded at the posterior midline of CF-L/D embryos, and that posterior enhancement contributes to naturally occurring changes in the pattern of heart asymmetry. Accordingly, we propose a model for midline effects in CF compared with SF embryos that highlights enhanced Shh signaling at both the anterior and the posterior poles of the embryonic axis (Fig. 8). At the anterior midline, increased Shh signaling controls the development of optic primordia by modifying the expression of Pax6, Pax2 and Vax2 transcription factors (Yamamoto et al., 2004), whereas our current results suggest that Shh upregulation at the posterior midline impacts the size and ciliation of the KV, L-R *dand5* asymmetry, downstream Nodal laterality, and the asymmetric pattern of heart development. In addition to Shh, the expression of other signaling systems, namely FGF, WNT and BMP4, are modified in the anterior midline and contribute to trait changes in CF embryos (Pottin et al., 2011; Hinaux et al., 2016; Ren et al., 2018). Likewise, we found that *bmp4* expression is also expanded in the CF post-chordal plate, suggesting that multiple signaling pathways are active in a posterior center that is instrumental in mediating developmental changes between the two forms of *A. mexicanus*. The Shh-regulated *foxj1a* gene, which controls cilia development in the KV (Yu et al., 2008), is also upregulated at the posterior midline of CF embryos where it is likely to be responsible for increases in the number of DFCs, KV size, and the length of KV cilia. It will be interesting to determine whether variations in the strength of these signaling systems in the post-chordal region occur between different vertebrate species, and whether such variation could influence traits that develop along the posterior midline, including those related to the regulation of L-R asymmetry.

We discovered duplicated, or even triplicated, KVs, each aligned in parallel at the posterior end of the CF notochord. To our knowledge, multiple KVs or LROs have not been described in other teleost or vertebrate species. In *A. mexicanus*, multiple KVs were seen in about 10% of CF-L/D embryos, which show the highest levels of heart asymmetry reversal, and more rarely in about 2% of CF-D embryos, but not in SF-D embryos. It is also notable that each of the multiple CF KVs was associated with a posterior spur of the *shh*-expressing notochord, supporting a notochord contribution to KV induction, which has been proposed in zebrafish embryos from other types of evidence (Compagnon et al., 2014). The multiple KVs are probably a consequence of increases in the number and condensation foci of DFCs, the KV precursors. The formation of double KVs in SAG-treated SF-D embryos, but not in controls, suggests that increased Shh is responsible for multiple KV development. However, the relatively low frequency of multiple KVs in CF embryos precluded in-depth investigation of their possible relationship to changes in L-R organ asymmetry. Future development of methods to increase the number of embryos with multiple KVs should allow their influence on L-R determination and Nodal laterality to be elucidated.

This study has revealed multiple changes related to enhanced posterior midline development and L-R asymmetry in CF. These changes occurred primarily or exclusively in CF-L/D relative to CF-D embryos (Table 1), and are correlated with their natural change in L-R visceral asymmetry. First, we found that DFCs are more abundant in CF-L/D than in CF-D or SF-D embryos, and in some CF-L/D embryos DFCs tend to aggregate into multiple foci instead of a single focal point at the end of gastrulation, as in CF-D and SF-D embryos. DFC development is controlled by maternal factors in zebrafish (Moreno-Ayala et al., 2021); if this is also true in *Astyanax*, then enhanced Shh expression and DFC/KV development could be targets of the maternal effects responsible for controlling CF-L/D heart asymmetry (Ma et al., 2021a). Nodal is a regulator of DFC development in zebrafish (Compagnon et al., 2014), and it should be noted that increased *ndr1* expression levels have been also reported in early CF-L/D gastrulae (Ma et al., 2021a), which could explain the increase in DFCs. Thus, in future studies it will be important to determine whether increased Shh expression in the post-chordal plate is also a maternal effect. Second, we found that the KV lumen of CF-L/D embryos inflates to about twice the size of their CF-D or SF-D counterparts at the 13-15 somite stage. There is a size threshold for normal KV function in zebrafish embryos, and relatively modest increases in KV size do not affect L-R laterality (Gokey et al., 2016). In contrast, our studies reveal that CF-L/D embryos with large KVs tend to exhibit abnormal L-R *dand5* asymmetry (see below) and heart asymmetry. Therefore, when KV inflate to a very large size, such as in CF-L/D embryos, there may be downstream effects on visceral organ asymmetry. Third, we discovered ciliary modifications in the KVs of CF-L/D compared with most CF-D and SF-D embryos, which are potentially important in the regulation of L-R asymmetry. One modification is that cilia density is decreased as a result of having the same number of cilia in a larger KV. Another modification is that KV cilia are lengthened, which may be an effect of elevated expression of *foxj1a*, the master regulator of the ciliary developmental program (Yu et al., 2008; Stubbs et al., 2008). In zebrafish, the strength of L-R fluid flow and subsequent heart laterality is related to KV cilia length (Lopes et al., 2010). Accordingly, we propose that one or both of these changes could interfere with the counterclockwise ciliary beat responsible for generating strong leftward fluid flow in the KV and thus be responsible for modifications in CF L-R asymmetry. Direct studies on ciliary movements will be needed to shed more light on the role of ciliary modifications in CF KV function.

**Table 1.**
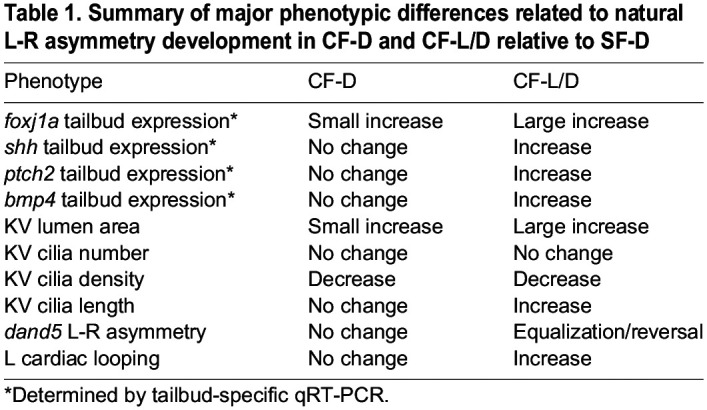
Summary of major phenotypic differences related to natural L-R asymmetry development in CF-D and CF-L/D relative to SF-D

Our results suggest that CF heart asymmetry reversal is controlled at least in part by the enhancement of a Shh signaling center in the post-chordal plate, which influences bilateral symmetry breaking by the KV. The findings supporting this conclusion are that an increase of Shh signaling in SF-D induced by SAG treatment can promote L-R asymmetric changes mimicking CF and a counter-acting inhibition of Shh signaling in CF-L/D caused by cyclopamine or SANT-1 inhibition can increase the levels of L-R asymmetric phenotypes to resemble those seen in CF-D and SF-D. The symmetry-breaking process in teleosts and other vertebrates with ciliated LROs includes downregulation of the Nodal antagonist *dand5* on the left side of the KV and activation of *spaw* expression in the left LPM (Long et al., 2003; Hashimoto et al., 2004; Smith and Uribe, 2021). We have shown that *dand5* asymmetry is abnormal in some CF-L/D embryos, which show either approximately equal levels of *dand5* expression on both the left and right sides of the KV or more expression on the left than the right side of the KV, a pattern that was mimicked by increasing Shh expression by SAG treatment in SF-D embryos and decreased by cyclopamine or SANT-1 treatment in CF-L/D embryos. These effects of Shh signaling on Dand5 asymmetry have not been described in other vertebrate species. However, it has been reported that Hedgehog signaling mediates changes in L-R asymmetry by downregulating Dand5 in the cephalochordate amphioxus (Hu et al., 2017; Zhu et al., 2020). Together, our results and the amphioxus studies reveal a potentially important role for the ciliary-driven Hedgehog-Dand5 axis in L-R symmetry breaking in chordates.

The mechanisms underpinning Shh-mediated equalization of *dand5* asymmetry in CF are unknown, and this result is difficult to reconcile with bilateral *spaw* expression in the CF-L/D LPM (Ma et al., 2021a). However, in addition to equalizing the Dand5 signal, Shh may also downregulate overall *dand5* expression and thus could activate the Nodal signal in both LPMs, although our current results are not clear on this issue. Nevertheless, an intriguing possibility for future consideration is that Shh signaling may regulate Dand5 asymmetry by affecting the Bicc1-Gcr4 RNA complex that degrades *dand5* mRNA on the left side of the LRO (Minegishi et al., 2021).

## MATERIALS AND METHODS

### Biological materials

*Astyanax mexicanus* SF and Pachón CF were raised in the laboratory in a constant flow aquatic system. In this study, we used SF families showing >95% D cardiac looping (SF-D), CF families with >90% D cardiac looping (CF-D), and CF families with 20-30% L-cardiac looping (CF-L/D) (Ma et al., 2021a). The fish were raised in the laboratory at 25°C on a 14-h light and 10-h dark photoperiod. Spawning was induced as described by Ma et al. (2021b), and embryos and larvae were raised at 23-25°C. Experimental protocols were conducted according to guidelines of the University of Maryland, College Park (IACUC #R-NOV-18-59; Project 1241065-1) and carried out in compliance with ARRIVE guidelines.

### Heart asymmetry analysis

Heart asymmetry was determined at 2-2.5 dpf in living larvae anesthetized with 2 mg/ml MS222 (Tricaine; Western Chemical Inc.) by visual inspection of cardiac tube looping from the ventral side under a Zeiss Axioskop compound microscope or in larvae fixed overnight with 4% paraformaldehyde (PFA) for immunostaining with the myosin heavy chain MF-20 antibody (Bader et al., 1982; Developmental Studies Hybridoma Bank) as described by Ma et al. (2021a). Embryos were classified as having right (D) looping, no (straight) looping, or left (L) looping cardiac tubes.

### Kupffer's vesicle analysis

The KVs of SF-D, CF-D and CF-L/D embryos were viewed laterally or dorsal-posteriorly by live imaging under a Zeiss Axioskop microscope at the 10-12, 13-15, 16-18 and 19-21 somite stages, and KV lumen perimeters were outlined and converted into areas using ImageJ software. To quantify KV cilia, 15-somite stage embryos were fixed overnight in 4% PFA at room temperature to preserve microtubules, then dehydrated through increasing concentrations of methanol to 100%, and stored at −20°C. For cilia immunostaining, the rehydrated specimens were washed three times in PBST (0.1% Tween-20 in PBS), blocked with 1% bovine serum albumin and 5% normal goat serum in PBST for 2 h, stained with anti-acetylated tubulin antibody (1:500; T7451, Sigma-Aldrich) in 1% bovine serum albumin at 4°C overnight, washed three times in PBST, and post-stained with Alexa Fluor-complex secondary IgG (Thermo Fisher Scientific, A32723, 1:500). *z*-stack images of the immunostained KVs were taken from their dorsal-posterior poles under a Zeiss LSM 980 Airyscan 2 laser scanning confocal microscope. KV cilia were counted manually and lengths were measured using ImageJ.

### Dorsal forerunner cell staining and quantification

The highly endocytotic DFCs were specifically stained with the vital fluorescent dye SYTO 11 (S7573, Invitrogen) as described by Cooper and D'Amico (1996). SF-D, CF-D and CF-L/D embryos were raised to the 60% epiboly stage, dechorionated by treatment with 0.2 mg/ml protease type XIV (P5147, Sigma-Aldrich) for 1 min at room temperature, incubated for 30 min with SYTO 11 diluted in DMSO to a final concentration of 15 µM, and then washed several times in water from fish culture system (fish system water). Control embryos were treated with 2% DMSO and then washed in system water. At the 70-80% epiboly stage, embryos were imaged using a Zeiss Axioskop compound microscope and fluorescent DFCs were quantified manually from photographic images.

### *In situ* hybridization

SF-D, CF-D and CF-L/D embryos were dechorionated by protease treatment (see above) or manual removal with forceps, then fixed in 4% PFA overnight, dehydrated in a series of increasing methanol concentrations to 100%, and stored at −20°C. RNA probes were prepared using oligonucleotide primers (Table S1) designed using sequence information from the *A*. *mexicanus* SF genome assembly (Warren et al., 2021) in the NCBI repository. *In situ* hybridization was carried out as described by Ma et al. (2014). After the completion of hybridization, the embryos were washed with PBST and incubated in BM Purple AP Substrate (Roche) for 4-48 h at room temperature in the dark. After the signal developed, the reaction was terminated by rinsing the embryos in PBS. The embryos were processed through an increasing glycerol series in PBS and photographed using a Zeiss Axioskop compound microscope.

### Tailbud isolation and RNA extraction

SF-D, CF-D and CF-L/D embryos were raised to the 13-15 somite stage and tailbuds were removed manually using Precision Glide metal needles (0.45 mm×16 mm, BD Biosciences) under a stereomicroscope. The isolated tailbuds were immediately immersed in TRI Reagent Solution (Life Technologies) and total RNA was extracted using the Direct-zol RNA MicroPrep kit (Zymo Research).

### Quantitative real-time RT-PCR

Total RNA from whole embryos or isolated tailbuds prepared as described above was treated with RNase-free DNase I (Zymo Research) to remove genomic DNA. cDNA was synthesized using the SuperScript IV VILO Master Mix and oligo (dT)_20_primers (Sigma-Aldrich). qRT-PCR was performed using TB Green Premix E×Taq II (Takara Bio) under the following cycling conditions: initial denaturation for 30 s at 95°C, then 40 cycles for 5 s at 94°C and 30 s at 60°C, and finally 5 s at 95°C, 1 min at 60°C, then raised to a final temperature of 95°C at increments of 0.11°C/s. The oligonucleotide primers (Table S2) for qPCR were designed using sequence information from the *A. mexicanus* SF genome assembly (Warren et al., 2021). The *rpl11* gene was used as a reference gene. The qRT-PCR reactions were performed using a LightCycler 480 (Roche). The ΔCt for each gene was calculated by subtracting the average Ct value of the reference gene. To compare gene expression, ΔΔCt was calculated by subtracting the average ΔCt of the control from the gene of interest. For graphical representation, the fold change was calculated as 2^−(ΔΔCt)^. The ΔΔCt values were used for statistical analyses.

### Increase of Sonic Hedgehog signaling in SF

The Shh pathway was increased by treating SF-D embryos with the cell-permeable Smoothened agonist SAG (11914, Cayman Chemical) (Stanton and Peng, 2010). A 15 mM SAG stock solution was prepared in DMSO. Beginning at 50-60% epiboly, embryos were dechorionated as described above and treated with 1 µM SAG or DMSO (control) for 8 h from 50-60% epiboly to the 13-15 somite stage, for 4 h from 50-60% epiboly to 90% epiboly, or for 4 h from 90% epiboly to the 13-15 somite stage. At the end of each treatment, embryos were washed with fish system water, then some embryos were processed for KV analysis, tailbud isolation for RNA extraction, and qRT-PCR as described above, or washed into fresh system water and raised until later stages to assay optic development. At 2-2.5 dpf, SAG-treated embryos and controls were processed for eye size measurements and detection of lens apoptosis, as described below, and the direction of cardiac looping, as described above. In addition, some SAG and control DMO-treated embryos were raised to about 3 months post-fertilization to determine long-term effects of Shh overexpression.

### Inhibition of Sonic Hedgehog signaling in CF

The Shh pathway was inhibited by treatment of CF-L/D embryos with 20 µM cyclopamine (11321, Cayman Chemical) or 2 µM SANT-1 (1974, Tocris) (Chen et al., 2002a, b). Stock solutions were prepared in 100% ethanol or DMSO, respectively, and inhibitor treatments were carried out for the same 8 h periods as described above for SAG. At the end of each treatment, embryos were washed with fish system water, and immediately fixed and processed for *in situ* hybridization as described above, or incubated until 2-2.5 dpf and processed for assaying cardiac looping.

### Eye measurement and lens apoptosis detection

SAG- and DMSO-treated SF embryos were raised until 2-2.5 dpf. Eye areas were measured by live imaging using a Zeiss Axioskop compound microscope and ImageJ software. Lens apoptosis was assayed by staining with 5 μg/ml Lysotracker Red DND 99 (Invitrogen) for 30 min in the dark (Ma et al., 2018). Stained larvae were anesthetized with MS222 (see above) and mounted on glass slides for imaging.

## Supplementary Material

10.1242/develop.202611_sup1 Supplementary information
