# Supplementary material for: Natural reversal of cavefish heart asymmetry is controlled by Sonic Hedgehog effects on the left-right organizer
Source: Development. 2024 Jul 18;151(14):dev202611. doi: 10.1242/dev.202611 (PMC11273321; doi:10.1242/dev.202611)
Supplement: Supplementary information [file develop-151-202611-s1.pdf]

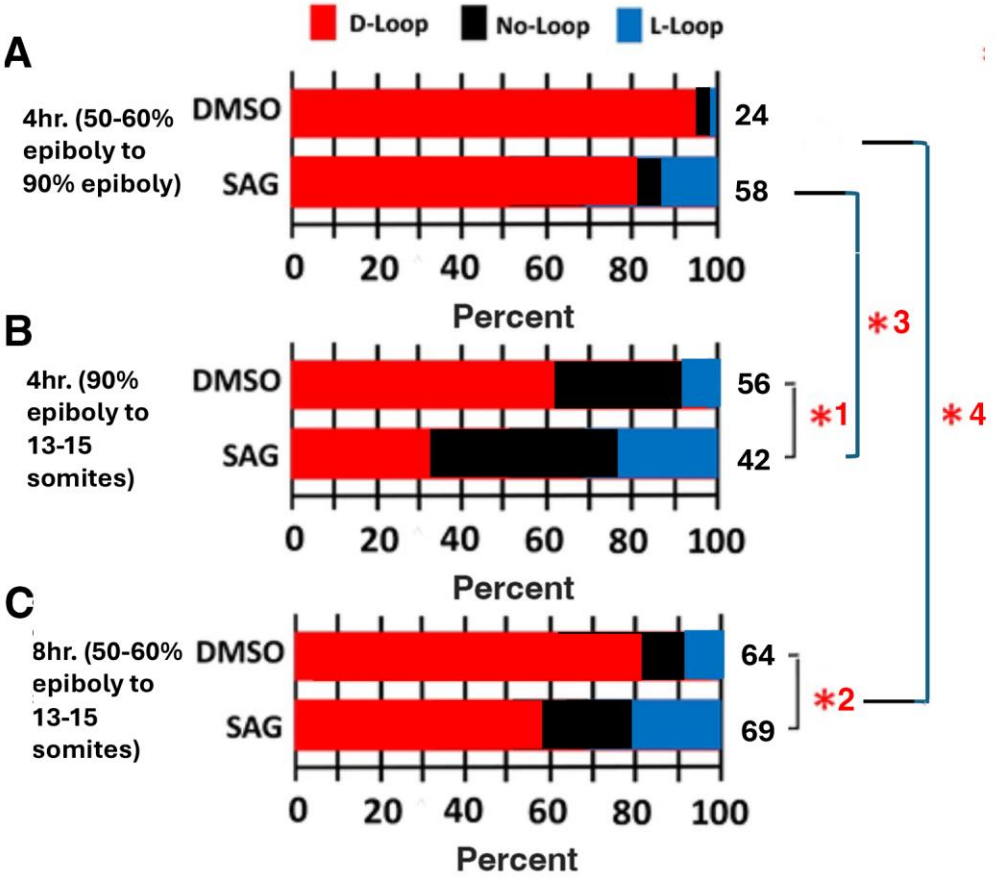

Fig. S1. Effects of SAG treatment duration on heart asymmetry in surface fish. A. A 4 hr. SAG treatment from 50-60% epiboly to 90% epiboly. B. A 4 hr. SAG treatment 90% epiboly to the 13-15 somite stage. C. An 8 hr. SAG treatment from 50-60% epiboly to the 13-15 somite stage. Asterisk 1: Chi^2 statistic = 8.7622; p = .012512. Asterisk 2: Chi^2 = 8.4463; p = .014652. Asterisk 3: Chi^2 = 26.2387; p < .00001. Asterisk 4: Chi^2 = 26.4423; p = .00026. No asterisk = no significance. Statistics by Chi^2 test.

Development • Supplementary information

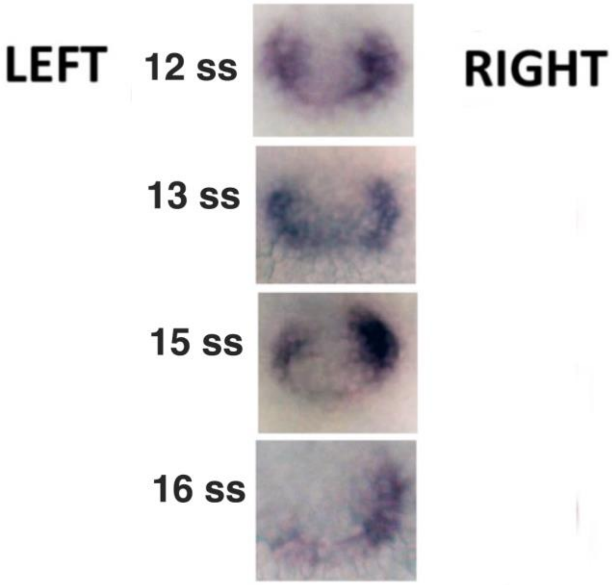

Fig. S2. Development of *dand5* asymmetry in surface fish. In situ hybridization showing *dand5* expression around KV from the 12 to16 somite (S) stages.

Table S1. Oligonucleotide primer sequences used to prepare RNA probes for in situ hybridization

Gene	Forward Primer	Reverse Primer
cldn5a	5'-GAAGGTGACGGCGTTCATCG-3'	5'-GAAGGTGACGGCGTTCATCG-3'
foxj1a	5'-TGCCGAAGCCACAATGTTTG-3'	5'-TGGTTCGACAAGAGCCACTC-3'
shha	5'-TATGAAGGCCGGGCCGTGGA-3'.	5'-CCGGGTACGACGTTGCTCGC-3'
bmp4	5'-CTCCCAGCACCACGTAACAT-3'	5'-CAGGTGTAGCACCTCCAAGG-3'
ptch2	5'-*GTCGATGCAGAACGCAGTGA*-3'.	5'-CAGGGTCGGCTGTGAATGTC-3'
c1orf127	5'-GGTGGTCGCACTGGAAGATG-3'.	5'-GGGTGACTTGTTCTCCATAGTCC-3'
dand5	5'-TTGTTCTGGTCGTGTTCCTGG-3'	5'-CGGCTCAGAAATACGCTGGG-3'
spaw	5'-TTTAACGTGACCGCTCTGCT-3'	5'-TGCATGTAGGCGTGATTGGT-3'

Table S2. Oligonucleotide primer sequences used for qRT-PCR

Gene	Forward Primer	Reverse Primer
foxj1a	5'-CTGCTACTTCCGCCATGCTG-3'	5'-CTTTCCTGGCTCGTCTTTCT-3'
shha	5'-AATTCCGAGCGCTTCAAGGA-3'	5'-AGCTTGTCTTTGCACCTCTGT-3'
bmp4	5'-GGAGCGCAAGAGGTCAACTT-3'.	5'-CCAGTCATGTATGGCCCGTC-3'
ptch1	5'-GTGTGTCCATCAACTCTTCTCAAA-3'	5'-GACACGCATAGGCAAGCATAA-3'
gli1	5'-ATCAACCAAGCAAAGGTCTGG-3'	5'- GGATGGGTTGTACATTGCTCTG-3'
nkx2.1	5'-GGAACCTGGGCAACATGAG-3'	5'-GAACCCATGAAACGAGAGATTGT-3'
dand5	5'-TCCCCTTCACCCAGCGTATT-3'	5'-GAGGTGCAGTGTCCGAAACA-3'
spaw	5'-TCAAACCCACCAATCACGCC-3'	5'-TAGAGAGCGAGCTGAGACGG-3'
rp21	5'-CATGCCTCACAAGTGCTACC-3'	5'-TTTGCCAGGATTTTGCCCTTG-3'

Development • Supplementary information
